# MND1 functions as a potential prognostic biomarker associated with cell cycle and immune infiltration in kidney renal clear cell carcinoma

**DOI:** 10.18632/aging.204280

**Published:** 2022-09-10

**Authors:** Jiayu Fang, Jing Zhen, Yiyang Gong, Yun Ke, Bidong Fu, Yike Jiang, Jing Xie, Yue Liu, Yongqi Ding, Da Huang, Fan Xiao

**Affiliations:** 1 Second Affiliated Hospital of Nanchang University, Nanchang, China; 2 Second College of Clinical Medicine, Nanchang University, Nanchang, China; 3 Department of Thyroid Surgery, Second Affiliated Hospital of Nanchang University, Nanchang, China; 4 Department of Anesthesiology, Second Affiliated Hospital of Nanchang University, Nanchang, China

**Keywords:** kidney renal clear cell carcinoma, MND1, biomarker, prognosis, bioinformatics analysis

## Abstract

Kidney renal clear cell carcinoma (KIRC) is a common and invasive subtype of renal tumors, which has poor prognosis and high mortality. MND1 is a meiosis specific protein that participates in the progress of diverse cancers. Nonetheless, its function in KIRC was unclear. Here, TIMER, TCGA, GEO databases and IHC found MND1 expression is upregulated in KIRC, leading to poor overall survival, and MND1 can serve as an independent prognostic factor. Moreover, enrichment analysis revealed the functional relationship between MND1 and cell cycle, immune infiltration. EdU and transwell assays confirmed that MND1 knockdown surely prohibited the proliferation, migration, and invasion of KIRC cells. Additionally, immune analysis showed that MND1 displayed a strong correlation with various immune cells. Interference with MND1 significantly reduces the expression of chemokines. TCGA and GEO databases indicated that MND1 expression is significantly related to two m6A modification related gene (METTL14, IGF2BP3). Finally, the drug sensitivity analysis revealed 7 potentially sensitive drugs for KIRC patients with high MND1 expression. In conclusion, MND1 can be used as a prognostic biomarker for KIRC and provides clues regarding cell cycle, immune infiltrates and m6A. Sensitive drugs may be an effective treatment strategy for KIRC patients with high expression of MND1.

## INTRODUCTION

Renal cell carcinoma (RCC), is a prevalent renal malignant tumor, ranking third in the most prevalent urinary malignancy in the world after prostate and bladder cancer [1]. Kidney renal clear cell carcinoma (KIRC) is RCC’s main pathological subtype [2]. KIRC has a high incidence rate and mortality, which seriously affects human life and health [3]. Although we have made tremendous progress in diagnosis, screening, surgery, as well as therapy, KIRC clinical results keep less than satisfactory [4]. About 30% of patients show postoperative metastasis or local recurrence, accompanied by a poor prognosis [5]. As we known, KIRC is a heterogeneous disease without a unique biomarker for individual therapy at present. Therefore, it is urgent to investigate the mechanism of KIRC and dig out an effective molecular marker for faster diagnosis and more accurate prognosis.

The existing KIRC biomarkers include bone morphogenetic protein 8A [6] and Cripto-1 [7], but their reliability and accuracy still need to be improved. Meiotic nuclear divisions 1 (MND1) is an important protein in meiosis. It boosts homologous chromosome pairing DNA double-strand break (DSB) repair during meiosis [8]. What's more, MND1 plays a role as DNA repair during vegetative cell growth [9, 10]. A few research findings have testified that meiotic factors could serve as effective tumor therapeutic as well as biomonitoring targets [11–14]. Several studies have found that MND1 may be a new target for tumor therapy, but they have not studied the role of MND1 in tumors in depth [15, 16]. MND1 can help to improve the proliferative ability of carcinoma cells [10, 17]. Furthermore, MND1 can promote circulatory progression in lung adenocarcinoma cells [18]. Its upregulation serves as an independent risk element for prognosis in LUAD sufferers. It also forms a positive feedback loop with KLF6 and E2F1 to regulate the cell cycle [19, 20]. Meanwhile, MND1 also endows cisplatin (DDP) resistance in LUAD [19]. However, the role and mechanism of MND1 in KIRC have not been reported yet and its relationship with prognosis remains unclear.

During this research, our group analyzed the expression of MND1 mRNA and protein in KIRC. And we explored the MND1 expression related to the prognosis of KIRC. Besides, we also delved into the connection between MND1 expression and cell cycle, tumor infiltrating immune cells, m6A, drug sensitivity in KIRC patients. Our results reveal the important function of MND1 in renal clear cell carcinoma and provide a potential link between MND1 and cell cycle, m6A, drug sensitivity, KIRC immune invasion and its underlying mechanism.

## MATERIALS AND METHODS

### Data collection and processing

KIRC gene expression profiles and corresponding clinical data were collected from the TCGA database (https://cancergenome.nih.gov) [21]. According to the gene expression features, our research contained 539 KIRC tumor samples and 72 normal samples. The file type was HTSeq-FPKM. We got the clinical information of 611 patients.

### Patients and tumor specimens

KIRC tissues and matched adjacent tissues were pulled together from 30 cases undergoing nephrectomy in the Second Affiliated Hospital of Nanchang University from June 1, 2017 to January 1, 2021. The patients gave informed consent to our collection of specimens. At the same time, the research ethics committee of the Second Affiliated Hospital of Nanchang University agreed the experiment.

### Cell lines and cell culture

The human KIRC cell lines Caki-1 Cells (No. TCHu135) was purchased from the National Collection of Authenticated Cell Cultures in China. All these cell lines were cultured in DMEM (Gibco, CA, USA) supplemented with FBS (Hyclone) to a final concentration of 10%, and cultured in a humidified incubator containing 5% CO2 at 37° C.

### TIMER database analysis

TIMER (1.0) (https://cistrome.shinyapps.io/timer), a consummate website, could be used to dissect the levels of immune invasion in various cancers [22]. Here, our group recognized the expression of MND1 in multiple cancers applying the “Diff Exp module”. Then the interrelation of MND1 together with immune infiltration in cancer was estimated using the “Gene module”. Moreover, we applied “SCNA module” to make a comparison between tumor infiltration levels among tumors and various somatic copy number changes in MND1. We assessed the differences between infiltration level for each SCNA category and the normal through a two-sided Wilcoxon rank-sum test. Finally, the correlations of MND1 with the markers of tumor infiltration immune cells in KIRC were verified using the “Correlation module”, joined with the Spearman’s rho value and predicted statistical implications.

### UALCAN database analysis

UALCAN (08/16/2021) is a user-friendly, comprehensive web portal which gives insight into TCGA gene expression data (http://ualcan.path.uab.edu) [23]. Our group used it to analyze MND1 expression in normal and KIRC cases on the basis of clinicopathological parameters, like cancer stage, age.

### Immunohistochemistry

The renal clear cell carcinoma tissue and matched corresponding 10% formalin-fixed and paraffin-embedded tissues were cut into 4um thick sections. After deparaffinization, rehydration, and microwave heating in sodium citrate buffer (10 mmol/L, pH 6.0) for 25 minutes to restore the antigen, the sections were sealed with goat serum for 30 minutes. Next, incubated the sections overnight with anti-MND1 polyclonal antibodies (RRID ab235395, Abcam, 1:50 dilution) at 4° C. Then, HRP-conjugated secondary antibody (Boster) was allowed to stand at room temperature for 2 hours. Subsequently, immunostaining was performed using a two-step method. Three pathologists scored staining intensity and the percentage of positive cells semi-quantitatively.

### LinkedOmics analysis

LinkedOmics (Oct 8, 2018) is a comprehensive data analyse platform (http://www.linkedomics.org/login.php) that can analyse multidimensional data within and across 32 kinds of cancer [24]. We tried to dig out the co-expressed genes linked to MND1 in the TCGA KIRC cohort through the results of analysis. These were shown by volcano plots and heat maps. These were founded by the LinkFinder. Pearson correlation coefficient was the concrete measurement of the association of results.

### Functional enrichment of differentially expressed genes

GO term and KEGG pathway enrichment analysis were conducted by the “clusterProfiler” package in R to identify the Gene Ontology (GO) annotations and pathways [25]. Pathways with P value < 0.05 was regarded as meaningful.

### Gene set enrichment analysis (GSEA)

GSEA can be applied for genome-wide expression profile analysis and interpretation built on biological knowledge [26]. We acted this analysis with Genomic Data Commons (https://portal.gdc.cancer.gov/). The parameters were established such as: gene set database: h. All. V7.4 Symbols. gmt (Hallmarks); number of permutations: 1,000. Those with a P value <0.05 and a false discovery rate (FDR) <0.25 were considered as indeed enriched pathways and genes.

### PPI network construction

The PPI network of the STRING (11.5) database (https://string-db.org/) [27] was applied to investigate the connection among the target genes. The parameter of medium confidence was set at 0.9. The top 200 hub genes were evaluated by Cytoscape 3.8.0 and its plug-in, MCODE (Molecular Complex Detection). And the selection criteria were as follows: Max depth=100, node score cutoff=0.2, K-core=2.

### Validation of the hub genes

Hub genes, highly connected with nodes in a module, have been proved to play an important role in function. The significance of the genes was measured by absolute value of the Pearson's correlation in our study. On the basis of the result, the top 200 genes with a confidence > 0.9 were uploaded to the STRING database to construct protein-protein interaction (PPI). Then, we used Cytoscape 3.8.0 and its plug-in, MCODE (Molecular Complex Detection) [28], to evaluate the top 200 genes. Furthermore, a standard for hub genes (yellow nodes) was set with a degree cut-off = 2, node score cut-off = 0.2, k-core = 2, and max. depth= 100, which were screened with MCODE. Totally, there were 48 genes scoring highest and initially defined as hub genes. Ultimately, after digging out their backgrounds, three genes, closely related to cell cycle, were regarded as “real” hub genes among these genes.

### GEPIA analysis

GEPIA (2017) is an online database serving for helping the analysis of RNA-seq data (http://gepia.cancer-pku.cn/) [29]. We explored the relationship between MND1 and the expression of particular markers correlated with cell cycle proteins of tumors. The Spearman method was chosen for the correlation coefficient analysis.

### GSCALite analysis

GSCALite (http://bioinfo.life.hust.edu.cn/web/GSCALite/) is a multifunctional genomics site, and we chose it to do pathway activity and drug sensitive analysis with Spearman test, based on a data set of TCGA KIRC. P<0.05 was considered statistically significant.

### EdU assay

A 5-ethynyl-20-deoxyuridine (EdU) assay kit was used to show cell proliferation ability. Briefly, cells were exposed to the indicated treatments. Roughly we plated 5*10^3^ cells/wells into 96-well plates and incubated 24h [30]. And we added 100 μl medium involving 50 μM EdU into each well. Cells were incubated for 2 h at 37° C, after fixation with 4% paraformaldehyde. Then staining the nuclei with Hoechst, and EdU solution was put into culture. Afterwards, results can be visualized by a fluorescence microscope [31].

### *In vitro* migration and invasion assays

After seeding for 48h, stably transfected cells were used for *in vitro* migration and invasion assays. When it came to migration, 6 x 10^4^ cells were plated in the upper chamber with serum-free medium. As for invasion, 1 x 10^5^ cells were placed in a Matrigel-coated chamber (BD Biosciences). After 24 hours (to examine migration) or 48 hours (to examine invasion) of seeding, the upper surface of the membrane was gently wiped to remove unmigrated cells. The cells migrated to the underside were fixed and stained with 0.1% crystal violet. Count cells in five random microscopic fields which use a light microscope with a DP70 CCD system (Olympus Corp., Japan).

### Western blot

Total protein extract was organized as mentioned in [32]. RIPA buffer (Beyotime, Shanghai, China) involving a protease and inhibitor mixture (Thermo Fisher Scientific, NY, USA) was managed for protein extraction on ice. After centrifugation, the protein concentration was studied by BCA protein assay kit (Thermo Fisher Scientific, Waltham, MA, USA). The same amount of protein was electrophoresed on SDS-PAGE and transferred to PVDF membrane. The primary antibody was then incubated overnight at 4° C and the membrane was washed 3 times with TBST. Simultaneously incubate with secondary antibody (anti-mnd1 polyclonal antibody (RRIDab235395, Abcam, 1:1000 dilution)) at room temperature for 2 hours. Finally, detect protein expression by electrochemiluminescence (ECL) method.

### Statistical analysis

The statistical analysis was mainly implemented by R software(version 3.6.3) with its packages named “survival”, “limma”, “ggplot2” and so on. Wilcoxon signed-rank test or Kruskal-Wallis test and logistic regression were used for comparing the MND1 expression levels among patients with different kinds of cancer, different kinds of organization, stage, grade, depth of tumor invasion, distant metastasis and gender. We used Kaplan–Meier method to portray the survival curve on the basis of log-rank test. The Cox proportional hazards regression model was applied to univariate and multivariate analyses. The T test was used to analyze the differential expression analysis of m6A-related genes between the high and low expression groups of MND1. During the entire study, the statistical significance threshold was P < 0.05.

## RESULTS

### MND1 is upregulated in human KIRC tissues and cell lines

**Figure 1:**
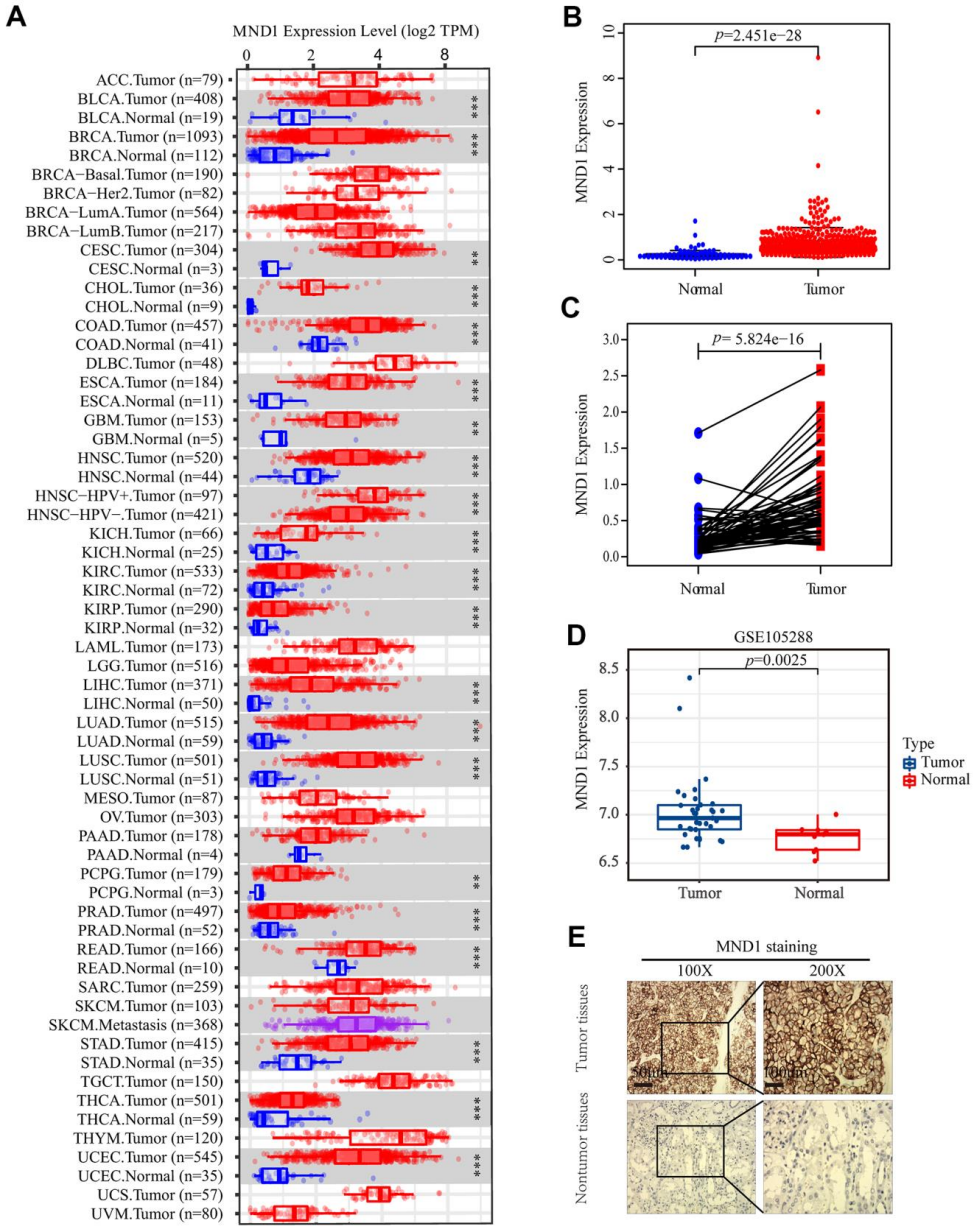
**The expression of MND1 in different datasets.** (**A**) The mRNA level of MND1 in 33 kinds of tumor types from TIMER. (**p<0.01, ***p<0.001). (**B**) Expression levels of MND1 were higher than non-tumor tissues in KIRC samples(*p*=2.451e-28). (**C**) MND1 expression of KIRC tissues and corresponding normal tissues downloaded from TCGA RNA-seq datasets(*p*=5.824e-16). (**D**) MND1 expression is significantly regulated in KIRC in the GSE105288. (**E**) Typical images of IHC in 30 pairs of KIRC tissues showing the protein expression of MND1 in KIRC and adjacent nontumor tissues.

To explore MND1 expression in various cancers, our study analyzed the TCGA-RNA sequence data in TIMER. It illustrated that MND1 was upexpressed in many types of cancer tissues than normal tissues. It was also true in KIRC (Figure 1A). Then, to analyze the amount of MND1 expression in normal tissues and tumor tissues, the Wilcoxon rank sum test was adopted to draw differential expression maps and paired differential expression map (Figure 1B, 1C). Both results showed that MND1 was obviously overexpressed in MND1 samples in the whole transcriptome sequencing (RNA-seq) dataset. To verify our finding, we used GEO dataset to analyze MND1 expression in KIRC, whose result showed MND1 mRNA expression were upregulated in LIHC (GSE105288 database) (Figure 1D). Besides, we lucubrated MND1 expression in KIRC clinical samples. We tested the protein level of MND1 via IHC, whose results confirmed that the protein expression level of MND1 was higher in 18 pairs of KIRC tissues (Figure 1E). In short, our results confirm the overexpression of MND1 in KIRC tissues.

### Relationship between MND1 expression and clinicopathological variables in KIRC

**Figure 2:**
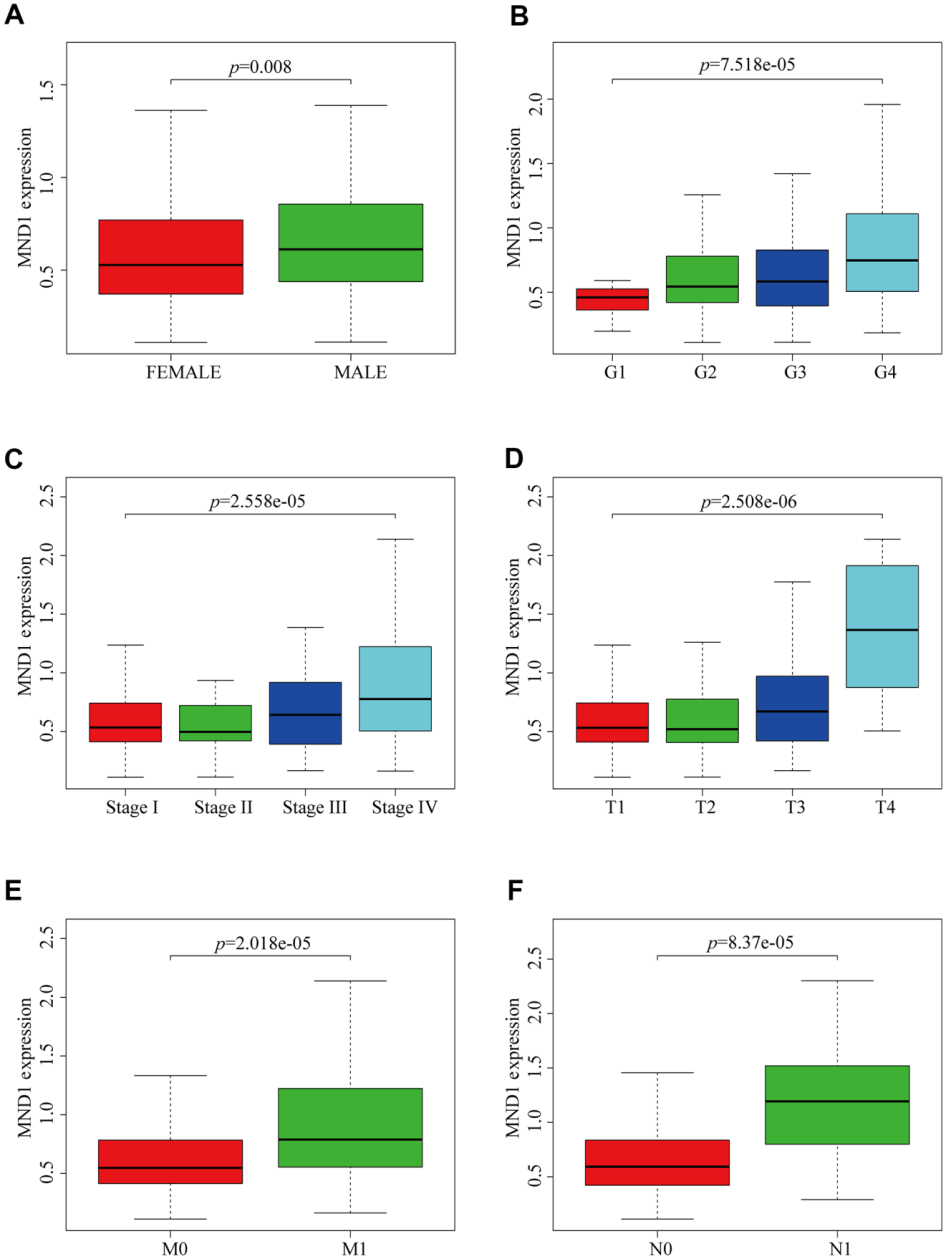
**MND1 expression is association with clinicopathological characteristics in patients with KIRC.** Increased MND1 expression was significantly with (**A**) Gender, (**B**) Grade, (**C**) Stage, (**D**) Tumor size, (**E**) Metastasis and (**F**) Node.

To explore the relationship between MND1 expression and KIRC sufferers’ clinicopathological features, based on TCGA database, we used Wilcoxon rank sum test to produce a series of related box-plots and used UALCAN to verify the result (Figure 2 and Supplementary Figure 1). Our finding suggested that there was a certain difference between males and females in the expression of MND1 (Figure 2A). Additionally, the expression of MND1 was closely related to tumor grade of KIRC and was positive correlated with it (Figure 2B). What's more, with the stage of KIRC getting higher, the expression of MND1 would also upregulate (Figure 2C). Also, the analysis result revealed that the expression of MND1 was positive correlated with T (Tumor size) (Figure 2D). Besides, we analyzed the relationship between MND1 and M (Metastasis). We found that MND1 had a higher expression in tumor tissue where distant metastasis occurred (Figure 2E). Finally, we also found that MND1 expression increased in tumor tissue with lymph node metastasis (Figure 2F). Subsequently, we verified with UALCAN and got the same result (Supplementary Figure 1). Afterward, on the purpose of analyzing the relationship between MND1 expression and poor clinicopathologic variables, we further adopted logistic regression. And the above consequences suggested that high MND1 expression was notably related to lymph node metastasis (OR=7.19 for N0 vs. N1), distant metastasis (OR=2.59 for M0 vs. M1) a high histologic grade (OR = 5.11 for G1 vs. G4), and gender (OR = 1.51 for Female vs. Male) (Supplementary Table 1). The above results confirmed that MND1 expression is closely correlated with clinicopathological characteristics.

### MND1 expression is an independent prognostic factor which is correlated with poorer prognosis of renal clear cell cancer patients

**Figure 3:**
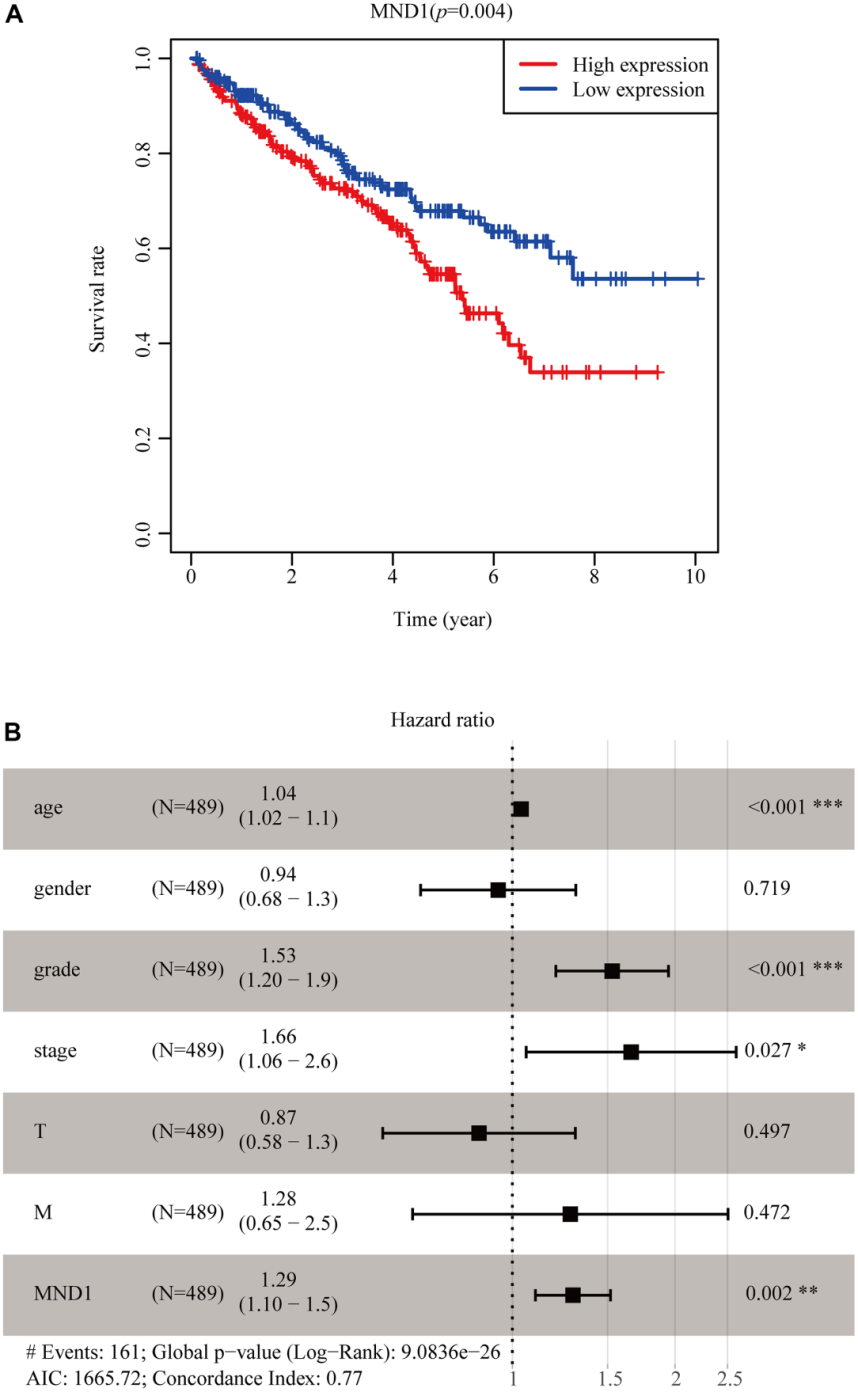
**MND1 expression in tumor tissues is associated with poor survival in KIRC patients.** (**A**) Associations with overall survival and the expression of MND1 in TCGA patients. (**B**) Multivariate Cox analysis of MND1.

In order to screen out the connections between MND1 expression and prognosis in TCGA patients with KIRC, we used Kaplan Meier survival method for survival analysis. The outcome proclaimed that in contrast to the low expression of MND1 (*p* = 0.004), the high MND1 expression predicted a poorer prognosis (Figure 3A). In addition, we constructed the ROC curve to detect sensitivity and specificity to predict one-year survival, three-year survival, and five-year survival of KIRC patients, The AUC of the ROC curve is significant, (one-year AUC:0.558, three-year AUC:0.532, five-year AUC:0.536), which indicates that the expression of MND1 can availably predict the survival time of patients (Supplementary Figure 2). Then we further dig out the connections between MND1 expression and clinical characteristics. The univariate Cox analysis indicated MND1 is substantially correlated with Overall survival (OS). The multivariate cox analysis exposed the variables of age, grade, and MND1 could regard as an independent predictive marker for the prognosis of sufferers with KIRC (Table 1). The forest map also reflects this point (Figure 3B). In summary, our results indicate that MND1 expression can be used as an independent prognostic parameter, and cases with elevated MND1 expression tend to be associated with a worse prognosis.

**Table 1:** Univariate and multivariate COX regression analysis of key genes.

**Variable**	**Univariate analysis**				**Multivariate analysis**		
	**HR**	**95%CI**	**P-value**		**HR**	**95%CI**	**P-value**
age	1.033	1.019-1.047	**<0.001**		1.038	1.023-1.054	**<0.001**
gender	0.931	0.675-1.284	0.663		0.941	0.676-1.310	0.719
grade	2.293	1.854-2.836	**<0.001**		1.530	1.203-1.945	**0.001**
stage	1.889	1.649-2.164	**<0.001**		1.659	1.061-2.594	**0.027**
T	1.941	1.639-2.299	**<0.001**		0.868	0.575-1.308	0.497
M	4.284	3.106-5.908	**<0.001**		1.280	0.653-2.506	0.472
MND1	1.353	1.184-1.547	**<0.001**		1.294	1.103-1.519	**0.002**

OS, overall survival; HR, hazard ratio; CI, confidence interval; TNM, Tumor Node Metastasis.

### Function enrichment analyses and co-expression genes of MND1 in KIRC

**Figure 4:**
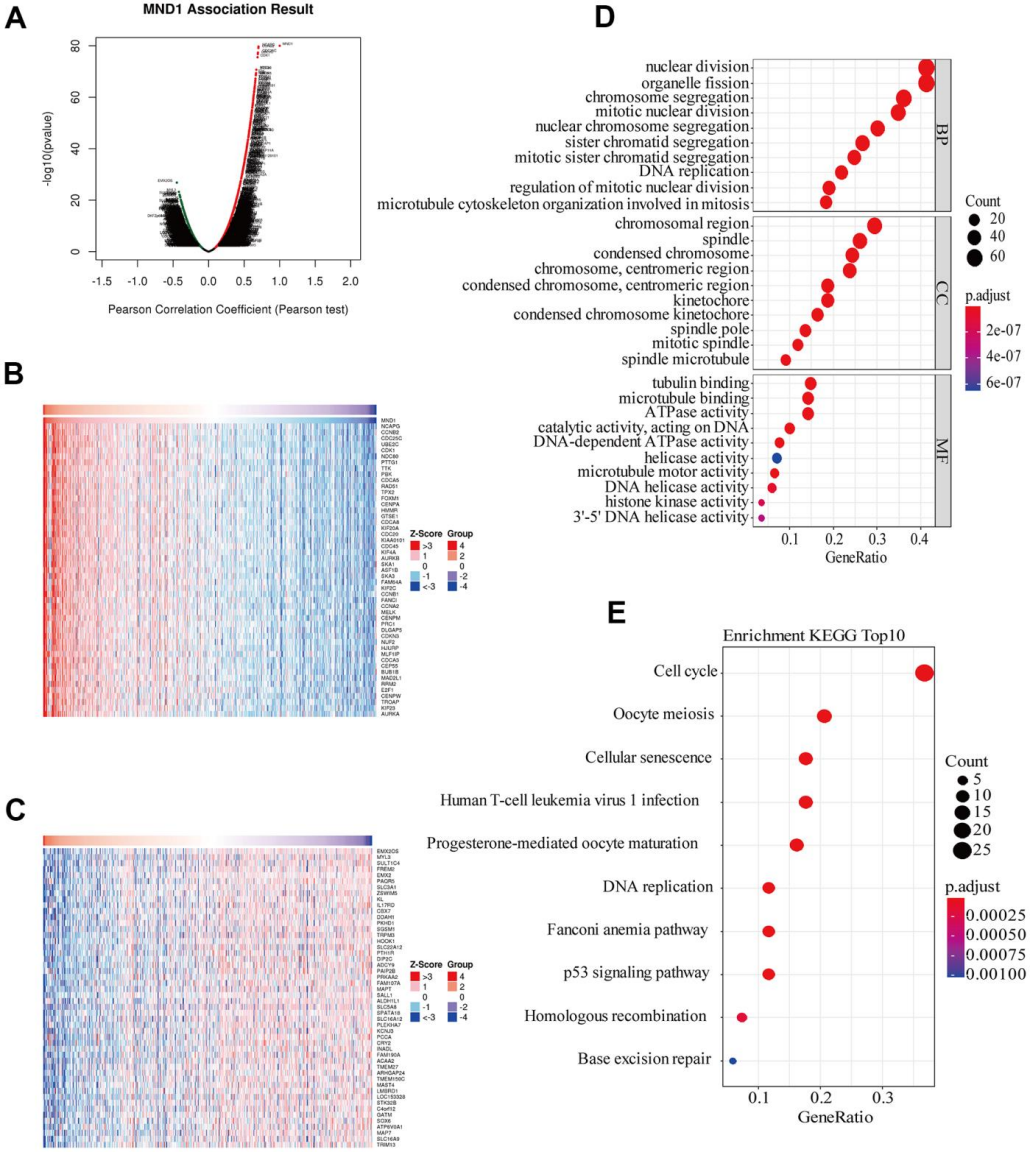
**Genes differentially expressed in correlation with MND1 and enriched GO annotations, KEGG pathways of MND1 correlated genes in KIRC.** (**A**) Pearson test was used to analyze association between MND1 and genes differently expressed in KIRC, red indicates positively correlated genes and green indicates negatively correlated genes. (**B**, **C**) The genes positively and negatively relative to MND1 in KIRC were showed by heat maps. (**D**), Enriched GO annotations of MND1 correlated genes in KIRC, including biological processes (BP), molecular function (MF), and cell component (CC) (*P*<0.05). (**E**) Significant KEGG pathways most associated with MND1.

To get to know the biological importance of MND1 in KIRC in depth, we applied the function module of LinkedOmics, aiming at testing the MND1 co-expression in KIRC. There were 7303 genes showing significantly positive correlation with MND1, significantly and dark red dots stand for them (Figure 4A). Meanwhile, 3930 genes which negatively correlated with MND1 were represented by dark green dots. 50 notable gene sets showing observably positive and negative correlation with MND1 were marked and listed in heatmaps (Figure 4B, 4C). The top 200 genes related most obviously to MND1 were extracted for enrichment analysis. We further explored the potential functional pathways based on the top 200 genes using ClusterProfiler R package. Functional enrichment and GO analysis suggested that MND1 was functionally related to cell cycle, DNA replication (Figure 4D). In addition, KEGG pathway analysis demonstrated an enrichment and crosstalk of the top 200 genes in P53 signaling pathway, cell cycle, oocyte meiosis. Cellular senescence, DNA replication, homologous recombination, mismatch repair, immune-related gene terms, containing human T-cell leukemia virus 1 infection (Figure 4E).

**Figure 5:**
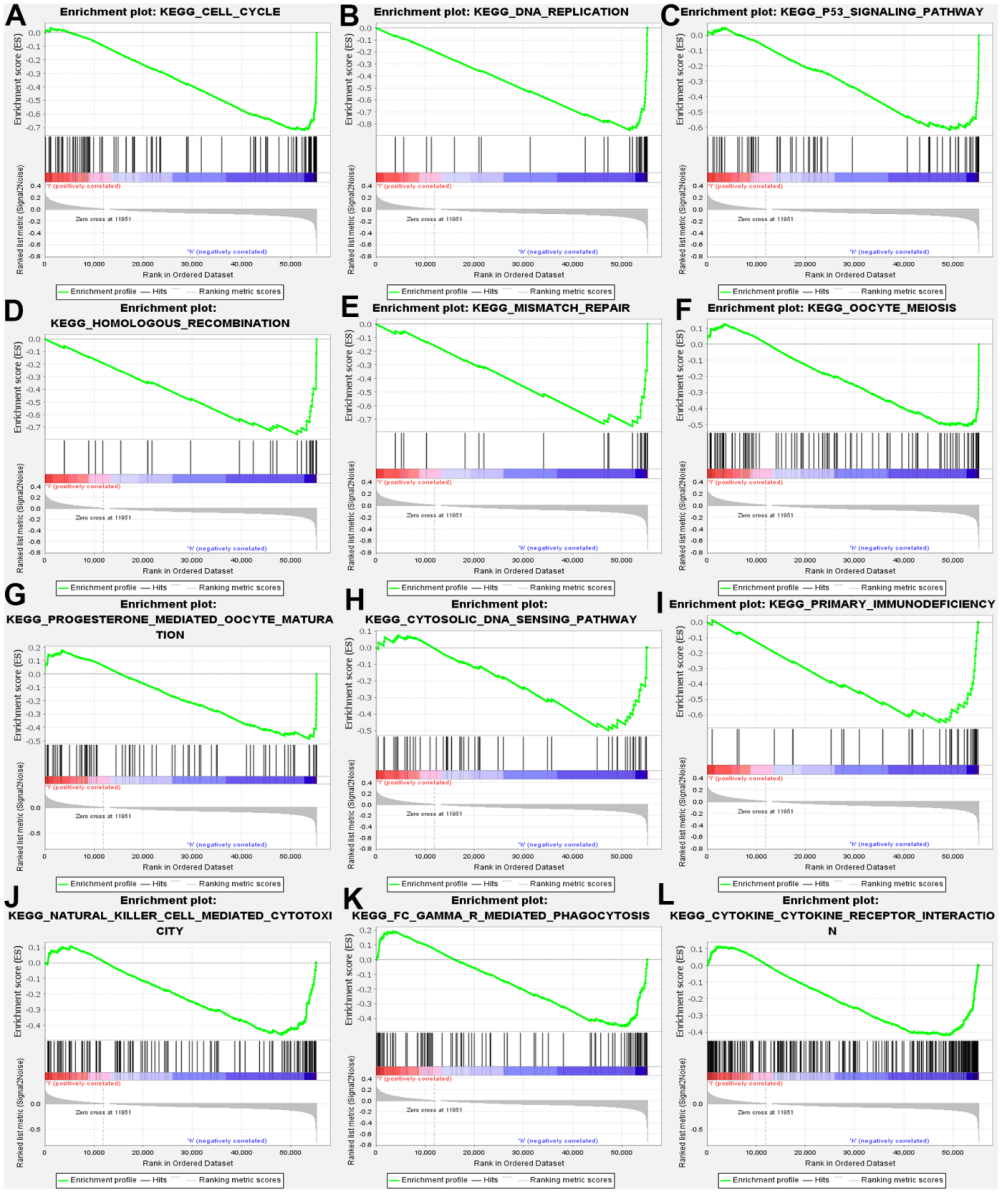
GSEA used to validate the gene signatures, including negative regulation of (**A**) CELL_CYCLE, (**B**) DNA_REPLICATION, (**C**) P53_SIGNALING_PATHWAY, (**D**) HOMOLOGOUS_RECOMBINATION, (**E**) MISMATCH_REPAIR, (**F**) OOCYTE_MEIOSIS, (**G**) PROGESTERONE_MEDIATED_OOCYTE_MATURATION, (**H**) CYTOSOLIC_DNA_SENSING_PATHWAY, (**I**) PRIMARY_IMMUNODEFICIENCY, (**J**) NATURAL_KILLER_CELL MEDIATED_CYTOTOXICITY, (**K**) FC_GAMMA_R_MEDIATED_PHAGOCYTOSIS, (**L**) CYTOKINE _CYTOKINE_RECEPTOR_INTERACTION.

Furthermore, GSEA was conducted, aiming at searching for KEGG pathways, which exposed that cell cycle, DNA replication, homologous recombination, mismatch repair, oocyte meiosis. Progesterone mediated oocyte maturation, Cytosolic DNA sensing pathway. In addition, the results confirmed the primary immunodeficiency, natural kill cell-mediated cytotoxicity, FcγR mediated phagocytosis, and cytokine receptor interaction (Figure 5). Above results demonstrated that MND1 is correlated with the cell cycle and immune-related pathways in KIRC.

### Correlation of MND1 expression with cell cycle

**Figure 6:**
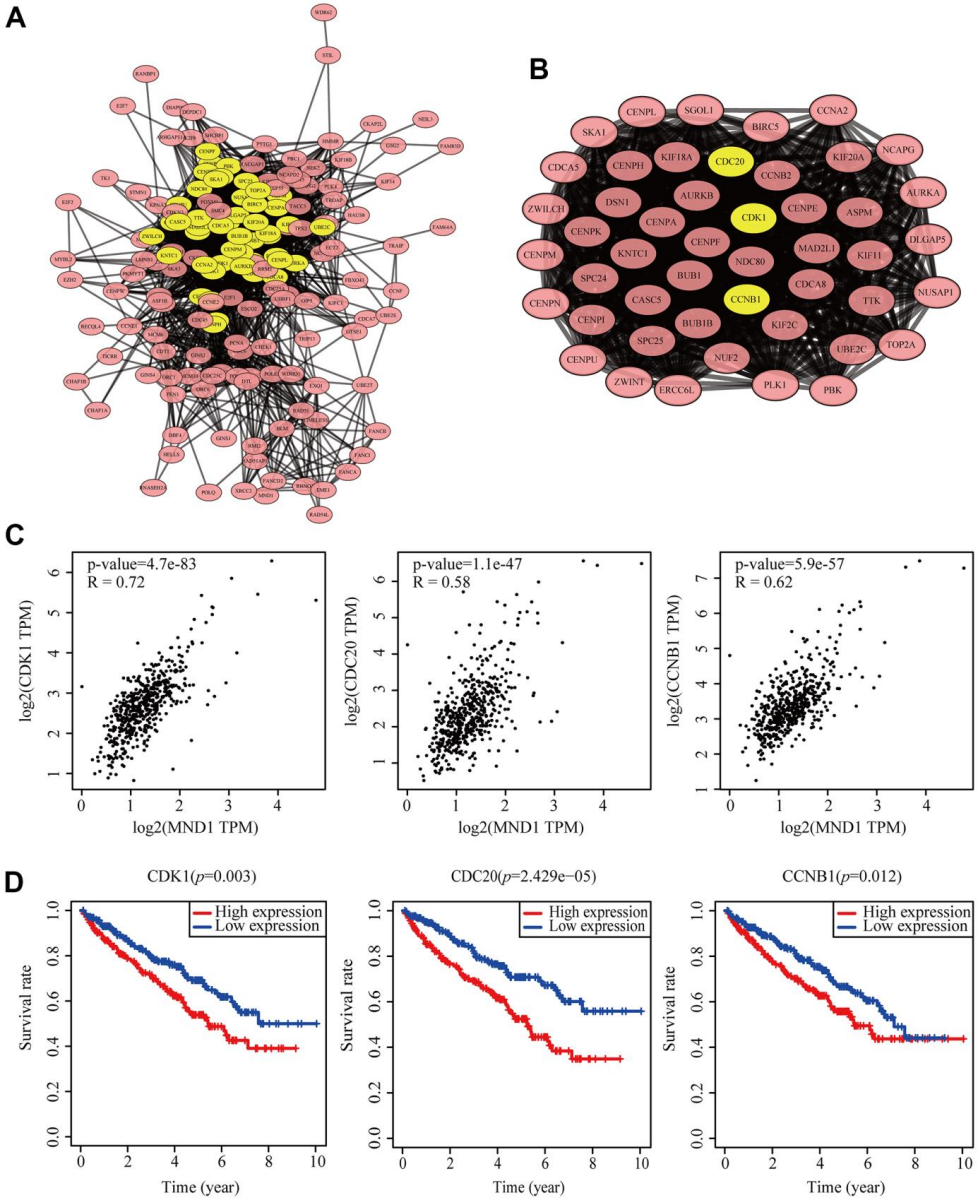
**Protein–protein interaction network of related gene (Top200) and analysis of hub genes in KIRC.** (**A**) Protein–protein interaction based on (PPI) network (**B**), MCODE analysis indicating the hub genes CDK1, CDC20, CCNB1, highlighted in yellow. (**C**) Correlation between MND1 and the mRNA expression of CDK1, CDC20 and CCNB1 in KIRC determined using GEPIA. (**D**) Prognosis analysis of correlational genes.

To dig into the functions of MND1 engaged, we analyzed the STRING database carefully. And then, we chose the top 200 co-expressed genes to make protein-protein interaction (PPI) network on the basis of it. Furthermore, we used Cytoscape (MCODE plug-in) to build the most significant module, marked in yellow (Figure 6A, 6B). According to the results obtained above, we could know that the module with higher scores consisted of CDK1, CDC20, and CCNB1. These three genes were regarded as the hub genes. At the same time, we discovered that there was a high correlation coefficient between MND1 and these three genes through GEPIA analysis (0.58<Spearman's correlation<0.71). Moreover, with the MND1 mRNA expression increasing, the mRNA expression of CDK1, CDC20 as well as CCNB1 in KIRC exhibited slightly upregulation (Figure 6C). Besides, we have done prognosis analysis of these genes with Kaplan-Meier Survival Method, which indicated that all of these three genes were oncogenes that were associated with poor prognosis (Figure 6D). Since CDK1, CDC20 and CCNB1 are known to be closely correlated to the cell cycle [33–38], with the support of the above analysis results, we inferred that the effect of MND1 on the prognosis of KIRC may be related to the cell cycle.

### MND1 knockdown inhibits KIRC cell proliferation *in vitro*

**Figure 7:**
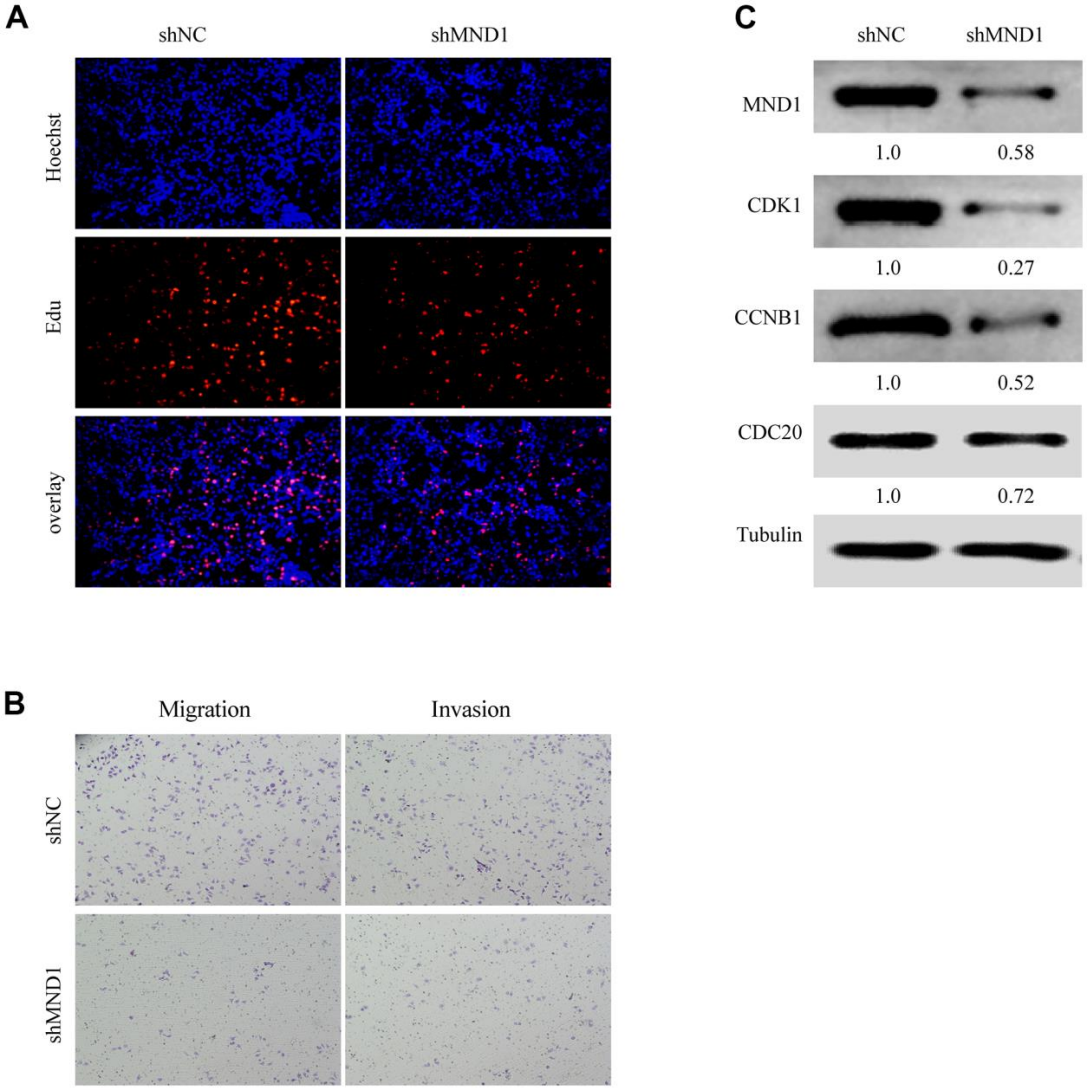
**MND1 promotes proliferation and migration of KIRC *in vitro*.** (**A**) Proliferation capacity for KIRC cells treated with shMND1 or shNC was detected by EdU and cell clone formation assays. (**B**) Migration and invasion capacity for KIRC cells treated with shMND1 or shNC was detected by Transwell separately. (**C**) The result of Western blot showed the protein expression of CDK1, CDC20, and CCNB1 was interfered with MND1.

We have confirmed that MND1 was closely related to the cell cycle. To delve the relationship between MND1 and KIRC cell proliferation, invasion, as well as migration, we further transfected Caki-1 cells with MND1-siRNAs and si-NC. The EdU assays revealed that knockdown of MND1 obviously cut down the proliferation of KIRC cells (Figure 7A). Transwell assays indicated that knockdown of MND1 dramatically reduced the invasion and migration ability of KIRC cells (Figure 7B). Moreover, we performed three Western blot experiments to confirm that MND1 downregulation significantly inhibited the expression of CDK1, CDC20, and CCNB1 (Figure 7C). These results indicate that MND1 might affect the proliferation, invasion, as well as migration ability of KIRC cells through the cell cycle.

### Correlation between MND1 expression and major infiltrating immune cells in KIRC

**Figure 8:**
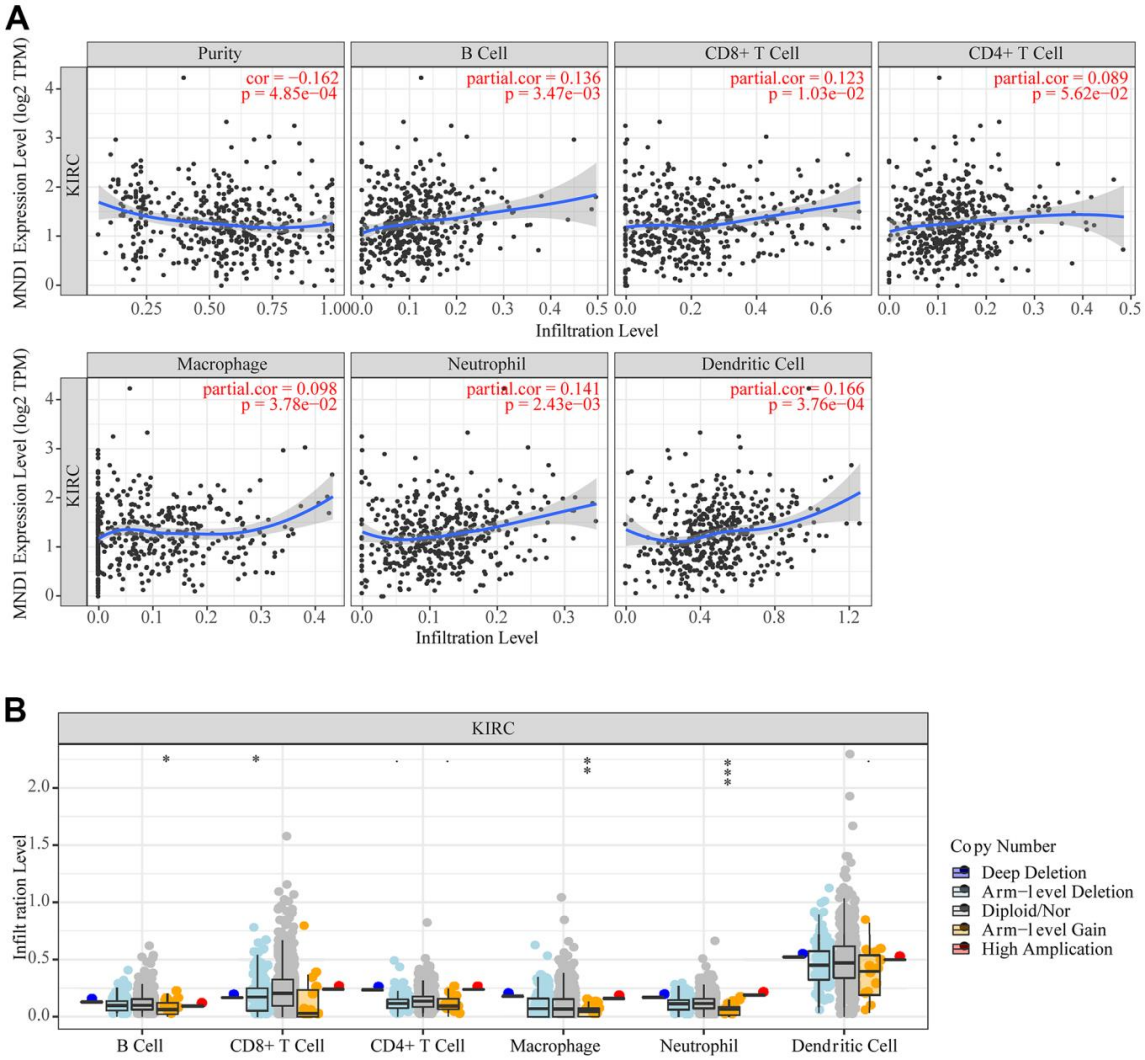
**The correlation between MND1 and immune infiltration level in KIRC.** (**A**) The correlations between MND1 expression and the immune infiltrations of tumor purity, B cell, CD8+ T cell, CD4+ T cell, macrophage, neutrophil, and dendritic cell. (**B**) The comparison of tumor-infiltration levels in KIRC with different somatic copy number alterations for MND1. SCNAs (somatic copy number alterations) are defined by GISTIC 2.0, including deep deletion (−2), arm-level deletion (−1), diploid/normal (0), arm-level gain (1), and high amplification (2). P-value Significant Codes: 0 ≤ *** < .001 ≤ ** < .01 ≤ * < .05 ≤. < .1.

Correlational researches have proved that the occurrence and development of a variety of cancers and their prognosis depend on the immune cell infiltration’s quantity and activity [39, 40]. Meanwhile, the results of GO, KEGG, and GSEA suggested that MND1 was related to immune infiltration. Therefore, to find out the correlation between MND1 expression and immune infiltration in KIRC, we adopted the tool of TIMER to analyze. The results showed a negative correlation between the levels of MND1 expression with the tumor purity of KIRC samples and significant correlations with different types of immune cells, including B cell (r=0.136; p=3.47e-03), CD8+T cell (r=0.123; p=1.03e-02), Macrophage (r=0.098; p=3.78e-02), Neutrophil (r=0.141; p=2.43e-03), and Dendritic cell (r=0.166; p=3.76e-04) (Figure 8A). Additionally, there was a remarkable correlation between MND1 CNV and infiltrating levels of B cell, CD8+ T cell, Macrophage, and neutrophil (Figure 8B). Thus, our work demonstrated MND1 regarding Tumor Purity as well as immune infiltration level in KIRC.

### Relationship between MND1 and immune cell gene markers

**Figure 9:**
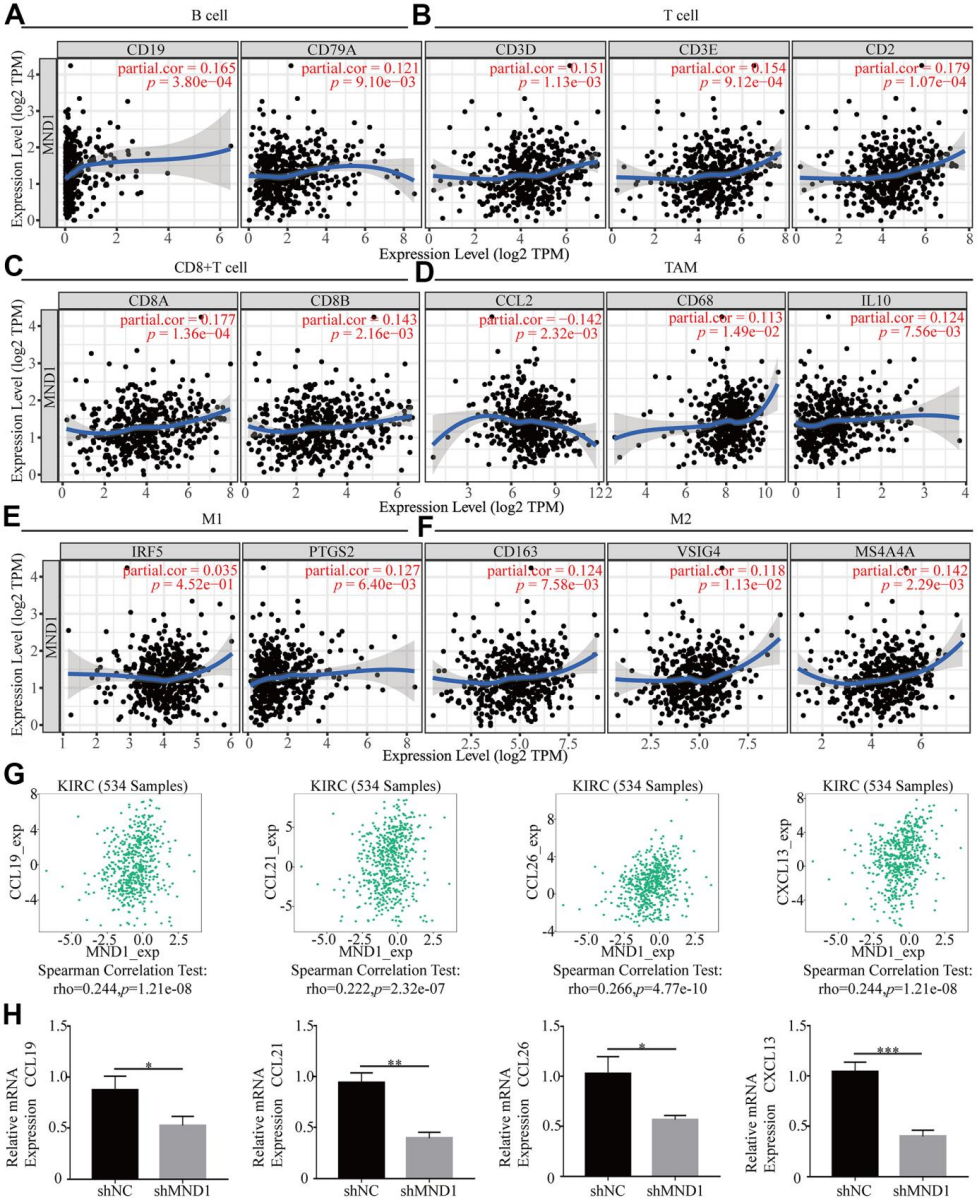
**The association of MND1 with immune cell gene makers in KIRC.** Relationship between MND1 and various gene markers of (**A**) B cells, (**B**) T cell (general), (**C**) CD8+ cell, (**D**) TAM, (**E**) M1 macrophage and (**F**) M2 macrophage in KIRC. (**G**) The association between MND1 and KIRC-related chemokines; (**H**) Immune-related chemokines expression in MND1 silenced KIRC cells. *p < 0.05, **p < 0.01, ***p < 0.001.

In order to delve into MND1’s potential relationship with infiltrating immune cells, our group tested the correlation between MND1 and multiple genetic markers for immune cells in TIMER. After adjusting for tumor purity, the MND1 expression level was obviously relevant to 16 out of 33 immune cell markers in KIRC (Table 2). Because not only B cell, T cell (general), but also CD8+ T cell, macrophages were mostly related immune cell types with MND1 expression, the connection between MND1 and immune marker sets of these cells were further investigated through TIMER. MND1 was positively related to some specific immune cell gene markers. It included B cell, T cell (general), CD8+ T cell, TAM, M1, M2 (Figure 9A–9F). Furthermore, MND1 was also closely linked to the KIRC-related chemokines including CCL19, CCL21, CCL26, and CXCL13. It could be known from the results that MND1 was positively associated with these chemokines (Figure 9G). Therefore, we had reasons to speculate that the high expression of MND1 can promote the proliferation, invasion and metastasis of tumor tissues through increasing the expression of some chemokines. However, it still needed further experiments to verify. Besides, we did the immune-related chemokines expression in Caki-1 Cells. As shown in Figure 9H, CCL19, CCL21, CCL26, and CXCL13 were all downregulated in MND1 shNC cells. The result verified our finding above that MND1 could affect immune cell infiltration partly by regulating these chemokines expression. In conclusion, these results proclaimed that MND1 was related to tumor cell infiltration in KIRC.

**Table 2:** Relationship between MND1 and gene marker sets of different immune cells using the TIMER database.

**Descriplion**	**Gene markers**	**KIRC**			
		**None**		**Purity**	
		**Cor**	**p**	**Cor**	**p**
**B cell**	CD19	0.188	**1.18E-05**	0.165	**3.80E-04**
	CD79A	0.141	**1.12E-03**	0.121	**9.10E-03**
**T cell (general)**	CD3D	0.173	**6.22E-05**	0.151	**1.13E-03**
	CD3E	0.173	**5.86E-05**	0.154	**9.12E-04**
	CD2	0.203	**2.41E-06**	0.179	**1.07E-04**
**CD8+ T cell**	CD8A	0.180	**2.87E-05**	0.177	**1.36E-04**
	CD8B	0.151	**4.86E-04**	0.143	**2.16E-03**
**Monocyte**	CD86	0.140	**1.19E-03**	0.125	**7.00E-03**
	CSF1R	0.105	**1.49E-02**	0.079	8.87E-02
**TAM**	CCL2	-0.120	**5.70E-03**	-0.142	**2.32E-03**
	CD68	0.112	**9.75E-03**	0.113	**1.49E-02**
	IL10	0.154	**3.50E-04**	0.124	**7.56E-03**
**M1**	IRF5	0.032	4.54E-01	0.035	4.52E-01
	PTGS2	0.153	**3.77E-04**	0.127	**6.40E-03**
**M2**	CD163	0.131	**2.46E-03**	0.124	**7.58E-03**
	VSIG4	0.139	**1.31E-03**	0.118	**1.13E-02**
	MS4A4A	0.142	**1.04E-03**	0.142	**2.29E-03**
**Neutrophils**	CEACAM8	-0.017	6.88E-01	-0.001	9.79E-01
	ITGAM	0.068	1.19E-01	0.033	4.86E-01
	CCR7	0.158	**2.58E-04**	0.149	**1.37E-03**
**Natural killer cell**	KIR2DL1	-0.020	6.46E-01	-0.020	6.76E-01
	KIR2DL3	-0.023	5.90E-01	-0.009	8.40E-01
	KIR2DL4	0.085	5.01E-02	0.078	9.57E-02
	KIR3DL1	-0.063	1.45E-01	-0.030	5.24E-01
	KIR3DL2	-0.039	3.71E-01	-0.035	4.57E-01
	KIR3DL3	0.051	2.35E-01	0.040	3.89E-01
**Dendritic cell**	HLA-DPB1	0.060	1.69E-01	0.040	3.86E-01
	HLA-DQB1	0.009	8.42E-01	0.004	9.35E-01
	HLA-DRA	0.083	5.41E-02	0.069	1.40E-01
	HLA-DPA1	0.091	**3.52E-02**	0.078	9.47E-02
	CD1C	0.053	2.18E-01	0.037	4.30E-01
	NRP1	0.036	4.09E-01	0.024	6.07E-01
	ITGAX	0.098	**2.43E-02**	0.076	1.01E-01

### Relationship between MND1 expression and m6A modification in KIRC

**Figure 10:**
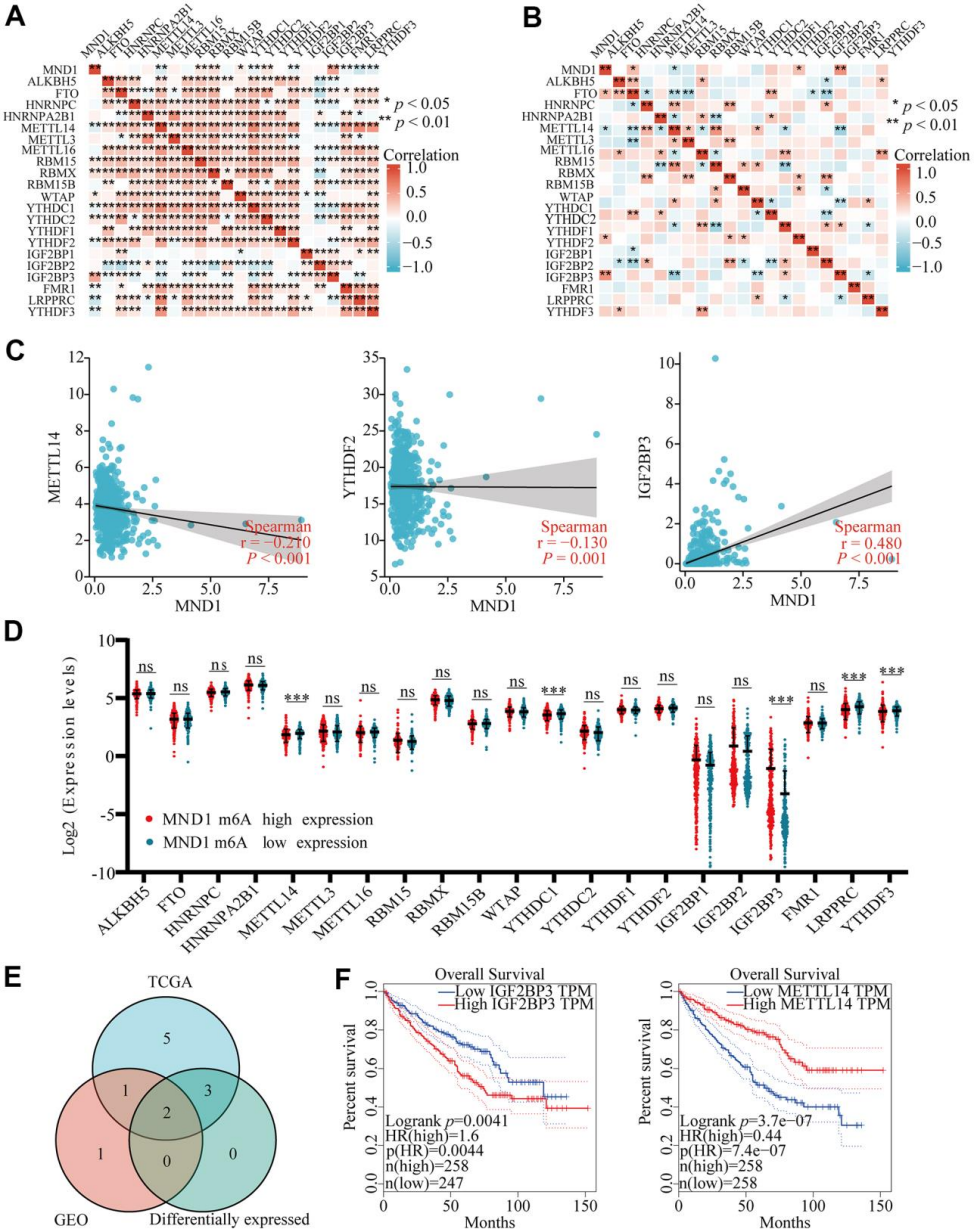
**Correlations of MND1 expression with m6A related genes in KIRC.** (**A** **B**) TCGA KIRC data sets and GSE105288 data sets analyzed the correlation between the MND1 and the m6A related genes expression in KIRC. (**C**) Draw a scatter plot to show the correlation between the MND1 and the glycolysis related genes expression, include METTL14, YTHDC1, and IGF2BP3. (***p<0.001). (**D**) The differential expression of glycolysis related genes between high and low MND1 expression groups in KIRC tumor samples. (**E**) Venn diagram showed both expression correlation and differential expression of genes, including ENO1, HK2, LDHA, LDHB, PGK1 and SLC2A1. (**F**) Kaplan-Meier curve of IGF2BP3 and METTL14.

Modification of m6A plays a significant role in the development of KIRC. By analyzing GSE105288 and TGGA KIRC data, we examined the correlation between MND1 expression and expression of 20 m6a related genes in KIRC, and the expression of MND1 significantly positively correlated with RBM15, RBMX, YTHDC2, IGF2BP3 and negatively related to METTL14 and YTHDF2 in TCGA KIRC data sets (*p* < 0.01) (Figure 10A). Furthermore, MND1 expression significantly negatively correlated with METTL14 (*p* < 0.05) and positively correlated with IGF2BP3 (*p* < 0.01) in the GSE105288 dataset (Figure 10B). The scattering plot showed the association between the expression of the genes related to MND1 and m6A (Figure 10C). We divided TCGA samples in two group according to the expression of MND1. We tried to exam the differential expression of genes related to m6A between high and low MND1 groups. As shown in Figure 10, the m6A modification was not the same between high and low groups with the MND1 expression in KIRC (Figure 10D). Compared to the group of low expression, the expression of METTL14 in the high expression group of MND1 were reduced and the expression of IGF2BP3 in it were increased (*P* < 0.001). Both correlation and differential expression of genes were present by Venn's diagram, including METTL14, and IGFBP3 (Figure 10E). Then, we used Kaplan-Meier curve to reveal that the high expression of IGFBP3 (*p* < 0.01) and low expression of METTL14 (*p* < 0.001) were intensely associated with a poor prognosis of KIRC (Figure 10F). These results claim that the MND1 may be closely related to the m6A modification of KIRC, especially through its regulation with METTL14, and IGFBP3, which eventually influents the progression and prognosis of KIRC.

### Cancer pathway activity and drug sensitivity

**Figure 11:**
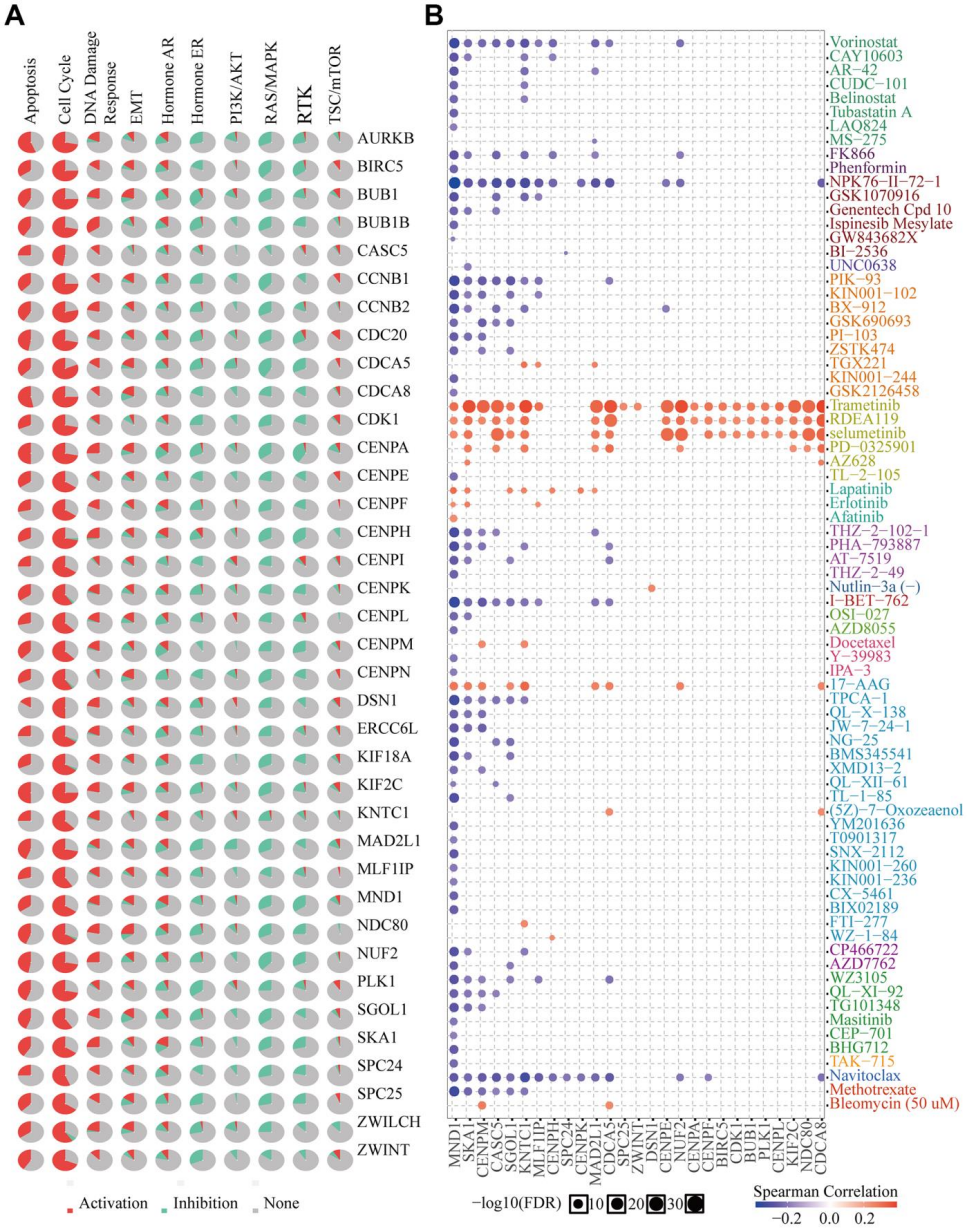
**Drug susceptibility analysis of hub genes.** (**A**) MND1-related Cancer pathway activity. (**B**) MND1-related drug sensitivity.

MND1 and 36 genes significantly correlated with MND1 in KIRC collected by PPI, were used for cancer pathway and drug susceptibility analysis. We used the GSCALite tool to evaluate the potential roles of these genes in the classic pathways of cancer. As shown in our result, these genes, especially MND1, could activate Apoptosis, Cell Cycle, DNA Damage Response, EMT, Hormone AR and inhibit Hormone ER, PI3K/AKT, RAS/MAPK, RTK pathways to play a regulatory role in the cancer process (Figure 11A). Moreover, KIRC cells with high-expressed MND1 level was resistant to 7 drugs or small molecules and 58-sensitive drugs (Figure 11B). These results showed innovative and optional therapeutic strategies for patients with highly expressed KIRC with MND1.

## DISCUSSION

RCC is a common renal malignant tumor, accounting for 2% of adult malignancies [41]. Among them, KIRC is the main pathological subtype of adult RCC [42]. Compared with patients with other subtypes of RCC, patients with KIRC have a higher rate of tumor recurrence and metastasis [43]. Although there have been a variety of clinical treatment strategies, the prognosis of KIRC patients is still not satisfactory due to the resistance of KIRC patients to radiotherapy and chemotherapy [44, 45]. In addition, a variety of biomarkers have been found in KIRC, such as bone morphogenetic protein 8A [6] and Cripto-1 [7], but their reliability is still controversial. Thus, exploring an effective biomarker is significant to enhance the treatment and prognosis of KIRC. Here, we determined that MND1 was a recent potential prognostic biomarker for KIRC and studied the association with MND1 and cell cycle, immune infiltration, m6A, drug sensitivity.

In our study, firstly, comprehensive bioinformatics were performed to reveal the expression and prognosis of MND1 in KIRC. The results showed that the mRNA and protein levels of MND1 were elevated in KIRC, which could lead to a poorer overall survival and prognosis of patients. Combined with the results of univariate multivariate Cox analysis it could also be known that MND1 was significantly correlated to overall survival (OS) and could be used as an independent prognostic factor.

Then, functional analysis was performed to deeply investigate the function and mechanism of MND1 in KIRC. Co-expressed gene analysis and GSEA analysis showed that MND1 is related to "nuclear division", "cell cycle", "DNA replication" and so on. In addition, "natural killer cell-mediated cytotoxicity" was related to MND1. The main conclusion drawn from our research was that MND1 was strongly associated with cell cycle and immune infiltration.

The cell cycle is a system which can be strongly regulated, enabling cell growth, genetic material replication, and cell division [46]. The progression from one cell cycle stage to another is driven by a mechanism composed of cyclin-dependent kinases (CDKs), cyclin proteins, and their catalytic partners [47]. All these mechanisms are often dysregulated in many tumors, leading to abnormal activation of cyclins [48]. Studies have shown that the abnormal activation of CDK1 in CDKs in various tumors leads to the development of tumor cells by regulating the cell cycle G2/M [49]. In addition, CDC20 is an essential cell cycle regulator, it can promote the development of tumor by inhibiting apoptosis and affect RCC formation [50]. CCNB1 takes a major part in regulating and forming a complex with CDK1 to promote cell cycle transition from G2 phase to mitosis [51]. And it promotes the occurrence and development of tumors in lots of cancers, for example, gastric cancer [52] and pancreatic cancer [53]. We used PPI to identify central genes, and it turned out that the central genes were CDK1, CDC20, and CCNB1, each of which played an important role in the cell cycle. In addition, GEPIA was used to evaluate the expression of MND1 and CDK1, CDC20, CCNB1. Moreover, the Kaplan–Meier survival method was used to investigate their prognosis. These showed that CDK1, CDC20, and CCNB1 were closely connected with MND1 expression. The high expression of CDK1, CDC20, and CCNB1 led to a low overall survival rate. This indicated that MND1's influence on the prognosis of KIRC may be related to the cell cycle.

Dysregulation of the cell cycle is the basis of the uncontrolled cell proliferation characteristic of the malignant phenotype [54]. Recent studies have shown that HOXA13 may promote KIRC proliferation through cell cycle arrest [55]. In addition, the disorder of the cell cycle is also related to the invasion and migration of tumors. Through EdU and transwell assays, we found that interfering with the expression of MND1 could greatly restrain the proliferation, invasion, and migration of KIRC cells. MND1 expression can also downregulate the protein level of CDK1, CDC20, and CCNB1. In summary, it showed that low expression of MND1 may inhibit cell proliferation, invasion, and migration through the cell cycle signaling pathway of KIRC cells.

More and more studies have found that immune responses could link to the clinical outcome in renal cell carcinoma. Tumor infiltrating immune cells (TIIC) form an ecosystem in the tumor microenvironment to manage the progression of cancer and show potential prognostic value [56]. A few RCC patients have high permeability of CD20 + B cells and poor prognosis [52]. Infiltration of CD4+ T cells contain RCC cell proliferation through regulating YBX1 [57]. High levels of active CD8+ T cells are related to the long-term prognosis of various cancers (including RCC) [58]. Tregs can effectively inhibit the proliferation of effector T cells in RCC [59]. In addition, M2 TAM can predict the clinical prognosis of KIRC patients [60]. Here, we confirmed the function of MND1 in KIRC immune infiltration. Additionally, we estimated the link between MND1 and immune cell infiltration in varying degrees. In recent years, many literatures reported that gene expression is closely related to tumor immune infiltration [61, 62]. Here owing to the expression level of MND1, we demonstrated that there are 6 types of tumor-infiltrating immune cells in KIRC tissues. Consistently, we found that MND1 is significantly associated with the gene marker set of B cell, T cell, CD8+ T cell, and macrophage immune cells. It is well known that chemokines play an important role in the recruitment and localization of immune cells in the tumor microenvironment [63, 64]. It was found through TISIDB that MND1 is positively correlated with CCL19, CCL21, CCL26 and CXCL13, and interference with MND1 can significantly reduce the expression of these chemokines. From the above, it demonstrates that MND1 acts a pivotal part in regulating the immune cell infiltration in KIRC.

m6A is the most common and abundant RNA epigenetic modification in eukaryotic cells, it can affect the occurrence and development of cancer by regulating cancer-related biological functions [65]. M6A is composed of “writers”, “readers” and “erasers”. METTL14 as “writers” and IGF2BP3 as “readers” play an important role in m6A modification [66]. Studies have found that IGF2BP3 can stabilize CDKN2B-AS1 through epigenetic activation of NUF2 transcription to drive the progression of KIRC malignant tumors [67]. The METTL14/BPTF axis enhances super enhancer and distal lung metastasis through glycolytic reprogramming in RCC [68]. In this study, we found that the expression level of MND1 was negatively correlated with METTL14 and YTHDF2, and was significantly positively correlated with IGF2BP3. We also found that in the MND1 high expression group, the expression levels of METTL14, YTHDC1, LRPPRC, and YTHDF3 decreased, while the expression levels of IGF2BP3 increased significantly. Finally, Kaplan-Meier curve analysis showed that KIRC patients with high expression of IGF2BP3 had a poor prognosis, and KIRC patients with low METL14 expression had a poor prognosis. We believe that MND1 is related to m6a, and may affect the methylation level of KIRC through IGF2BP3 and affect the progress of KIRC.

Finally, drug sensitivity analysis showed that the low expression of MND1 was sensitive to 58 drugs, and a positive correction was also observed between MND1 expression and drug sensitivity (Trametinib, RDEA119, Selumetinib, Lapatinib, Erlotinib, Afatinib, and 17−AAG). It indicates that seven sensitive drugs may be effective treatment strategies for KIRC patients with high MND1 expression.

This study has lots of limitations. Firstly, the data in the database is constantly updated and may affect the results obtained on the results website. We need to collect more clinical data for verification. Secondly, there are many ways to generate trust, and we will use more methods to verify the results.

To put it concisely, this article suggests that MND1 probably is a potential biomarker for poor prognosis in KIRC. MND1 is not only related to the cell cycle, it can regulate the proliferation, invasion and migration of KIRC. It may also play an important function in the microenvironment of KIRC by containing infiltrating immune cells. At the same time, MND1 is closely related to m6A, and the high expression of MND1 is sensitive to 7 drugs. These recommend that MND1 may function as a target for early clinical diagnosis and treatment, and at the same time provide a reference for further exploration of new cancer immunotherapy.

## Supplementary Material

Supplementary Figures

Supplementary Table 1
