# Supplementary material for: MND1 functions as a potential prognostic biomarker associated with cell cycle and immune infiltration in kidney renal clear cell carcinoma
Source: Aging (Albany NY). 2022 Sep 10;14(18):7416–42. doi: 10.18632/aging.204280 (PMC9550261; doi:10.18632/aging.204280)
Supplement: Supplementary Figures [file aging-14-204280-s001.pdf]

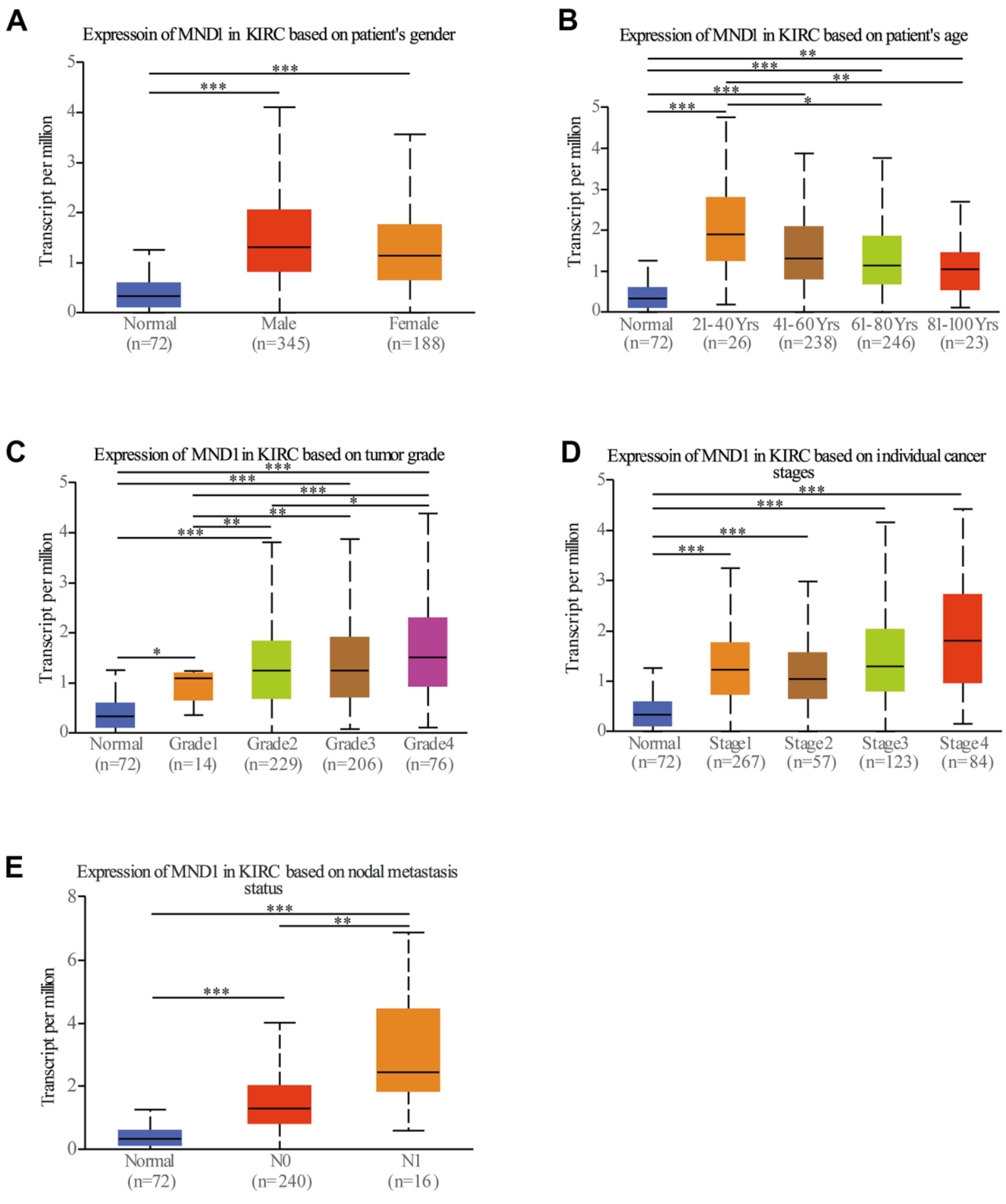

Supplementary Figure 1. Association between MND1 expression and clinicopathological characteristics in patients with KIRC(UALCAN). (**A**), Boxplot showing expression of MND1 in KIRC based on patients' gender. (**B**), Boxplot showing expression of MND1 in KIRC based on patients' age. (**C**), Boxplot showing expression of MND1 in KIRC based on tumor grade. (**D**), Boxplot showing expression of MND1 in KIRC based on cancer stages. (**E**), Boxplot showing expression of MND1 in KIRC based on node (***P<0.001, **P<0.01, *P<0.05).

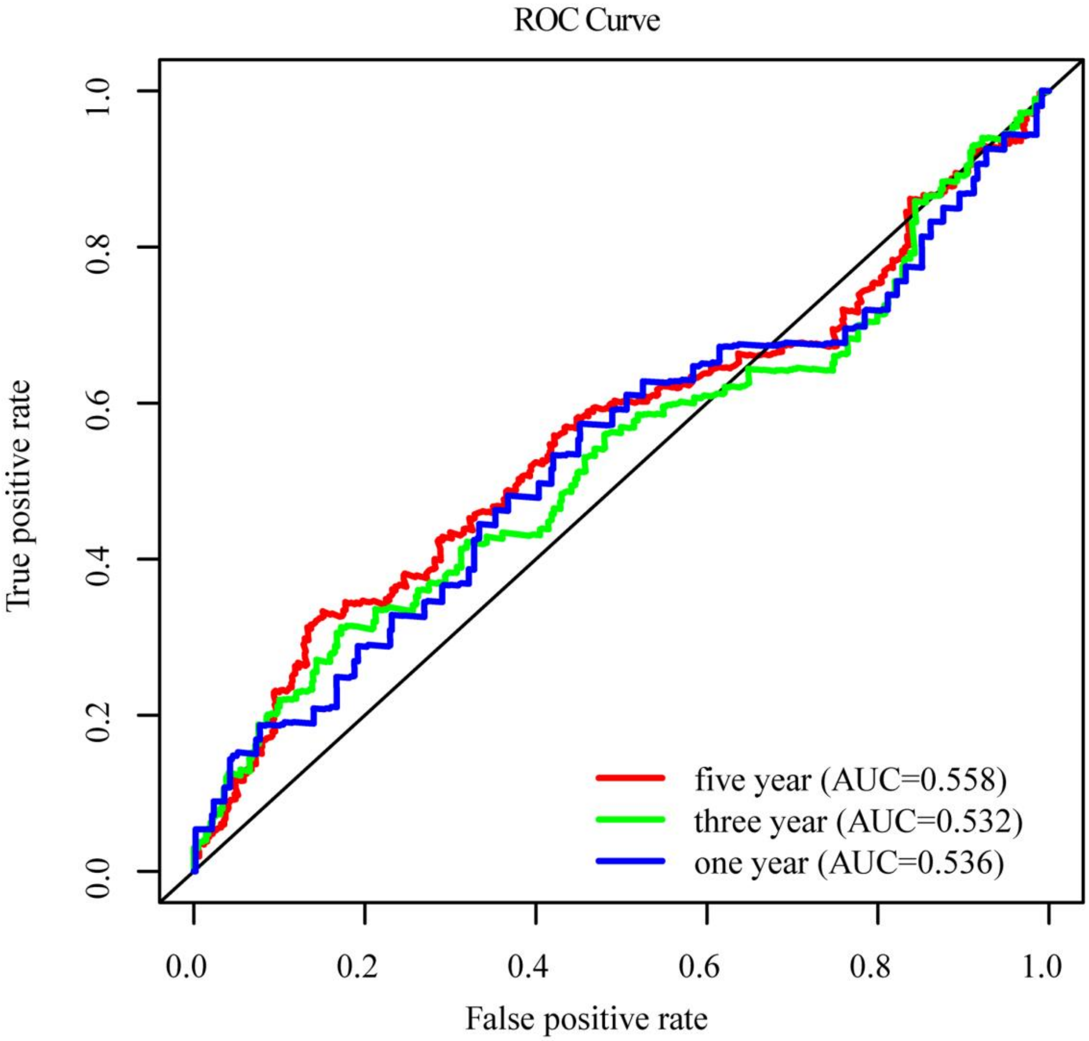

Supplementary Figure 2. ROC curve tests the accuracy of MND1 in predicting the survival rate of KIRC patients. ROC curves for the 1-, 3-, and 5-year survival according to the expression level of MND1. AUC, area under the curve; ROC, receiver operating characteristic.
