# Supplementary material for: MND1 functions as a potential prognostic biomarker associated with cell cycle and immune infiltration in kidney renal clear cell carcinoma
Source: Aging (Albany NY). 2022 Sep 10;14(18):7416–42. doi: 10.18632/aging.204280 (PMC9550261; doi:10.18632/aging.204280)
Supplement: Supplementary Table 1 [file aging-14-204280-s002.pdf]

## SUPPLEMENTARY TABLE

**Supplementary Table 1. MND1 expression associated with clinical pathological characteristics.**

| Clinical characteristics | Total (N) | Odds ratio in MND1 expression | P value |
|---|---|---|---|
| **Age** (≤ 60 vs. > 60) | 537 | 0.80(0.57-1.14) | 0.22 |
| **Gender** (Female vs. Male) | 537 | 1.51(1.05-2.17) | **0.02** |
| **Grade** (G1 vs. G4) | 92 | 5.11(1.35-33.35) | **0.04** |
| **Stage** (I vs. IV) | 326 | 0.62(0.33- 1.12) | 0.12 |
| **T stage** (T0 vs. T4) | 344 | 0.77(0.44-1.32) | 0.35 |
| **N stage** (N0 vs. N1) | 257 | 7.19(1.93-46.62) | **0.01** |
| **M stage** (M0 vs. M1) | 525 | 2.59(1.56-4.42) | **0.00** |

(logistic regression).

T, tumor; N, node; M, metastasis; Bold values indicate P-values<0.05.
